# Variable rates of hybridization among contact zones between a pair of topminnow species, *Fundulus notatus* and *F. olivaceus*

**DOI:** 10.1002/ece3.10399

**Published:** 2023-08-08

**Authors:** David D. Duvernell, Naznin S. Remex, Jeffrey T. Miller, Jacob F. Schaefer

**Affiliations:** ^1^ Department of Biological Sciences Missouri University of Science and Technology Rolla Missouri USA; ^2^ Molecular, Cellular, and Biomedical Sciences University of New Hampshire Durham New Hampshire USA; ^3^ Department of Biological Sciences University of Southern Mississippi Hattiesburg Mississippi USA; ^4^ Present address: Department of Molecular and Cellular Physiology Louisiana State University Health Sciences Center Shreveport Louisiana USA

**Keywords:** *Fundulus*, genomic cline, hybridization, introgression, phylogeography, reproductive isolation, Robertsonian fusion

## Abstract

Pairs of species that exhibit broadly overlapping distributions, and multiple geographically isolated contact zones, provide opportunities to investigate the mechanisms of reproductive isolation. Such naturally replicated systems have demonstrated that hybridization rates can vary substantially among populations, raising important questions about the genetic basis of reproductive isolation. The topminnows, *Fundulus notatus* and *F. olivaceus*, are reciprocally monophyletic, and co‐occur in drainages throughout much of the central and southern United States. Hybridization rates vary substantially among populations in isolated drainage systems. We employed genome‐wide sampling to investigate geographic variation in hybridization, and to assess the possible importance of chromosome fusions to reproductive isolation among nine separate contact zones. The species differ by chromosomal rearrangements resulting from Robertsonian (Rb) fusions, so we hypothesized that Rb fusion chromosomes would serve as reproductive barriers, exhibiting steeper genomic clines than the rest of the genome. We observed variation in hybridization dynamics among drainages that ranged from nearly random mating to complete absence of hybridization. Contrary to predictions, our use of genomic cline analyses on mapped species‐diagnostic SNP markers did not indicate consistent patterns of variable introgression across linkage groups, or an association between Rb fusions and genomic clines that would be indicative of reproductive isolation. We did observe a relationship between hybridization rates and population phylogeography, with the lowest rates of hybridization tending to be found in populations inferred to have had the longest histories of drainage sympatry. Our results, combined with previous studies of contact zones between the species, support population history as an important factor in explaining variation in hybridization rates.

## Graphical abstract

The topminnows, *Fundulus notatus* and *F. olivaceus*, co‐occur in drainages throughout much of the central and southern United States. We compared hybridization rates and genomic clines among nine contact zones. We found substantial variation among contact zones in hybridization rates, but no consistent patterns of variation in genomic clines across linkage groups. We did observe a relationship between hybridization rates and population phylogeography.

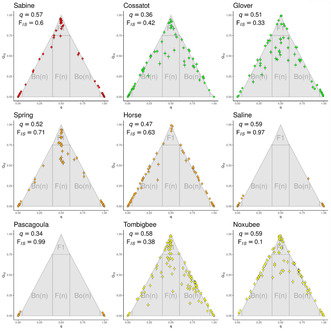

## 1 INTRODUCTION

Reproductive isolation is central to speciation, and interspecific contact zones or hybrid zones provide opportunities to evaluate the evolution of genetic isolation (Barton & Hewitt, 1989; Harrison, 1990). Species that exhibit broad, complex overlapping distributions, with replication of independent contact zones, provide particularly valuable opportunities to assess the generality of evolutionary outcomes of species contact. The best replicated systems are ones that control for phylogenetic history, in which the same pair of species have established multiple, independent contact zones (Culumber et al., 2011; Duvernell & Schaefer, 2014; Parchman et al., 2013; Schaefer et al., 2011; Westram et al., 2021; Zieliński et al., 2019). Such systems allow for elucidation of underlying processes leading to consistent patterns (Gompert et al., 2017; Harrison & Larson, 2016; Mandeville et al., 2015). However, genomic studies of hybridization in replicated systems have often instead demonstrated heterogeneity in patterns and prevalence of hybridization and backcrossing (Gompert et al., 2012; Kingston et al., 2017; Mandeville et al., 2015; Parchman et al., 2013). This raises an intriguing question about the impact of shared evolutionary history among populations in determining the evolution of reproductive isolation between reciprocally monophyletic species.

Even between reciprocally monophyletic species, variation in patterns of hybridization among populations may result from extrinsic factors, such as variable biotic or abiotic environmental factors among contact zones (Gompert et al., 2017; Nolte et al., 2009). Evolutionary processes, such as reinforcement, may be differentially impacted by variation in geographic scale and spatial structure of contact zones, shaped by environmental heterogeneity and underlying ecological gradients (Servedio & Noor, 2003). Alternatively, isolated populations may segregate variation for intrinsic hybrid fitness due to variation in genetic incompatibilities (Cutter, 2012; Gagnaire et al., 2013; Xiong & Mallet, 2022) that may come about as a result of complex phylogeographic histories (Zieliński et al., 2019). Geographically isolated populations may also exhibit variable and independent histories of secondary contact (e.g., Bossu & Near, 2009).

The complex genetic architecture of reproductive isolation may be revealed by population genomic studies (Westram et al., 2022). The assessment of genomic clines of single nucleotide polymorphisms (SNPs) provides a framework for evaluating reproductive isolation, and heterogeneity and uniformity in introgression across loci and chromosomes relative to genome‐wide admixture gradients (Gompert et al., 2017; Gompert & Buerkle, 2011). Genomic cline data provide a means of assessing the genome‐wide variability of patterns of hybridization and introgression, and for identifying genomic regions resistant to introgression (Gompert et al., 2017).

The role of chromosomal rearrangements, including inversions and Robertsonian (Rb) fusions, in causing reproductive isolation has long been debated (Baker & Bickham, 1986; Faria & Navarro, 2010; Navarro & Barton, 2003; Rieseberg, 2001). When Rb fusions are involved, reproductive isolation may be caused by underdominance, in which hybrid individuals have reduced fitness due to missegregation during meiosis (Baker & Bickham, 1986; Garagna et al., 2014; Sites & Moritz, 1987). The disruption caused by single Rb fusions may be minimal, allowing for fusions to become fixed in populations by drift or meiotic drive. However, the cumulative effect may be more disruptive in species differing by multiple accumulated fusions if the effects of individual fusions are multiplicative (Baker & Bickham, 1986; Chmátal et al., 2014; Garagna et al., 2014; Potter et al., 2015). Fusions may also contribute to reproductive isolation by facilitating coupling of loci under divergent natural selection and loci associated with reproductive barriers through linkage and suppression of recombination (Butlin & Smadja, 2018; Rieseberg, 2001). Support for this idea includes mapping studies that have demonstrated linkage between such loci (Berdan et al., 2021; Wellband et al., 2019). Despite the potential for Rb fusions to contribute to reproductive barriers, empirical studies have found that gene flow can be prevalent between species with such chromosomal differences (Horn et al., 2012; Potter et al., 2015).

## 2 TOPMINNOWS IN THE *FUNDULUS NOTATUS* SPECIES COMPLEX

In this study, we sought to add to a general understanding of genome‐wide patterns of reproductive isolation and the role of Rb fusions by investigating hybridization between two fish species in a naturally replicated system of contact zones. The *Fundulus notatus* species complex includes two topminnow species, the blackstripe topminnow (*F. notatus*) and black spotted topminnow (*F. olivaceus*), that are broadly distributed throughout much of the central and southern United States (Braasch & Smith, 1965; Thomerson, 1966). The species are of Pliocene age and reciprocally monophyletic (Duvernell et al., 2013, 2019). Populations of *F. notatus* cluster into a complex of four vicariant clades of Pleistocene age corresponding to gulf coastal drainage systems that include, the Western Gulf Slope, the Red River basin, the Mississippi River basin, and the Mobile River basin (Duvernell et al., 2019). In contrast, populations of *F. olivaceus*, with a very similar geographic distribution, exhibit comparatively limited range‐wide phylogeographic structure consistent with a relatively recent late Pleistocene range expansion (Duvernell et al., 2013, 2019). This has resulted in contact zones of varying ages and histories (Duvernell et al., 2019). Hybrid zone studies conducted with limited nuclear genetic markers (five nuclear restriction fragment length polymorphisms‐RFLPs) indicated that hybridization rates vary substantially across topminnow contact zones (Duvernell et al., 2007; Schaefer et al., 2011) and that hybrids may exhibit reduced fitness (Duvernell & Schaefer, 2014). Phylogenomics indicated historical intraspecific introgression among *F. notatus* clades, but did not find indications of interspecific introgression beyond zones of sympatry (Duvernell et al., 2019).

The divergence of *F. notatus* is marked by multiple Rb fusions. *Fundulus olivaceus* exhibits the ancestral condition of 24 pairs of chromosomes, while three of the four *F. notatus* clades exhibit 20 pairs of chromosomes that include four pairs of large metacentric chromosomes (Chen, 1971; Setzer, 1970). The fourth *F. notatus* clade, in the Mobile River basin, exhibits 24 pairs of chromosomes with only two pairs of large metacentric chromosomes (Black & Howell, 1978). So phylogeographic variation in numbers of fusions has resulted in species pairs that differ by either the presence of two or four Rb fusions in *F. notatus* relative to *F. olivaceus*. It is possible that karyotypic differences between *F. notatus* and *F. olivaceus* are foundational to reproductive isolation between the species. This could have served a role in facilitating shifting and expanding species ranges, and concomitant broad sympatry of the two species by enforcing postzygotic reproductive isolation upon secondary contact. This could be possible irrespective of whether chromosomal rearrangements were causative or coincidental agents of divergence and speciation.

In this study, we investigated nine contact zones at the genome scale. We had two specific objectives: First, we sought to evaluate and explore heterogeneity in hybridization rates across geographically isolated contact zones throughout the species overlapping ranges using high‐density genomic SNP markers. Second, we used genomic cline analyses of mapped linkage group markers to look for consistent patterns of genome‐wide heterogeneity in introgression and test the hypothesis that Rb fusions in *F. notatus* contribute to reproductive isolation, by evaluating whether populations exhibit distorted patterns of introgression among SNP markers on fused versus unfused linkage groups. We discuss the possible role of phylogeography, and population history in explaining geographic variation in patterns of hybridization.

## 3 MATERIALS AND METHODS

### 3.1 Draft genome assembly map

We wished to assign SNPs to linkage groups, and to determine which linkage groups in *F. olivaceus* were fused in *F. notatus*. This required separate scaffold assemblies using single F2 families for each species. First, we crossed one pair of *F. olivaceus* parents selected from a Gulf coastal population (Pascagoula) and an Ozark Highland population (Gasconade), respectively, to construct an F2 family of *F. olivaceus* progeny. We genotyped F2 progeny using genotype‐by‐sequencing (GBS) following Elshire et al. (2011). Genotype‐by‐sequencing libraries were constructed by the Elshire Group Ltd. using the EcoT22i restriction enzyme, and libraries were amplified with 18 PCR cycles. Libraries were multiplexed (188 individuals) and sequenced on the Illumina NovaSeq 6000 platform using two channel chemistry. The sequencing runs were 150 bp paired‐end. We demultiplexed and removed combinatorial barcodes using AX‐demux (Murray & Borevitz, 2018).

We aligned short reads from the *F. olivaceus* F2 family to a published *F. olivaceus* draft genome (Johnson et al., 2019), and then assembled those contigs into 24 *F. olivaceus* linkage groups following Miller et al. (2019). This scaffolded draft genome was used to generate mapped SNPs from reference‐aligned short‐read sequences for this study. Details regarding mapping are included in Supporting Information.

Second, to determine which *F. olivaceus* linkage groups were fused in *F. notatus*, we constructed an F2 family of *F. notatus* using parents from Western Gulf (Sabine) and Ouachita Highland (Glover) populations. Although these parents were members of different *F. notatus* clades, both populations exhibit *n* = 20 chromosomes with four Rb fusions. F2 progeny was genotyped following the same approach as *F. olivaceus*. We aligned short reads from the *F. notatus* F2 family to the *F. olivaceus* draft genome contigs (because an *F. notatus* draft genome was not available) and then assembled those contigs into 20 *F. notatus* linkage groups. The synteny of linkage groups between the two species was then established by aligning the *F. olivaceus* and *F. notatus* assemblies to each other using MUMmer 4.0 (Marçais et al., 2018).

### 3.2 Contact zone sampling

**FIGURE 1:**
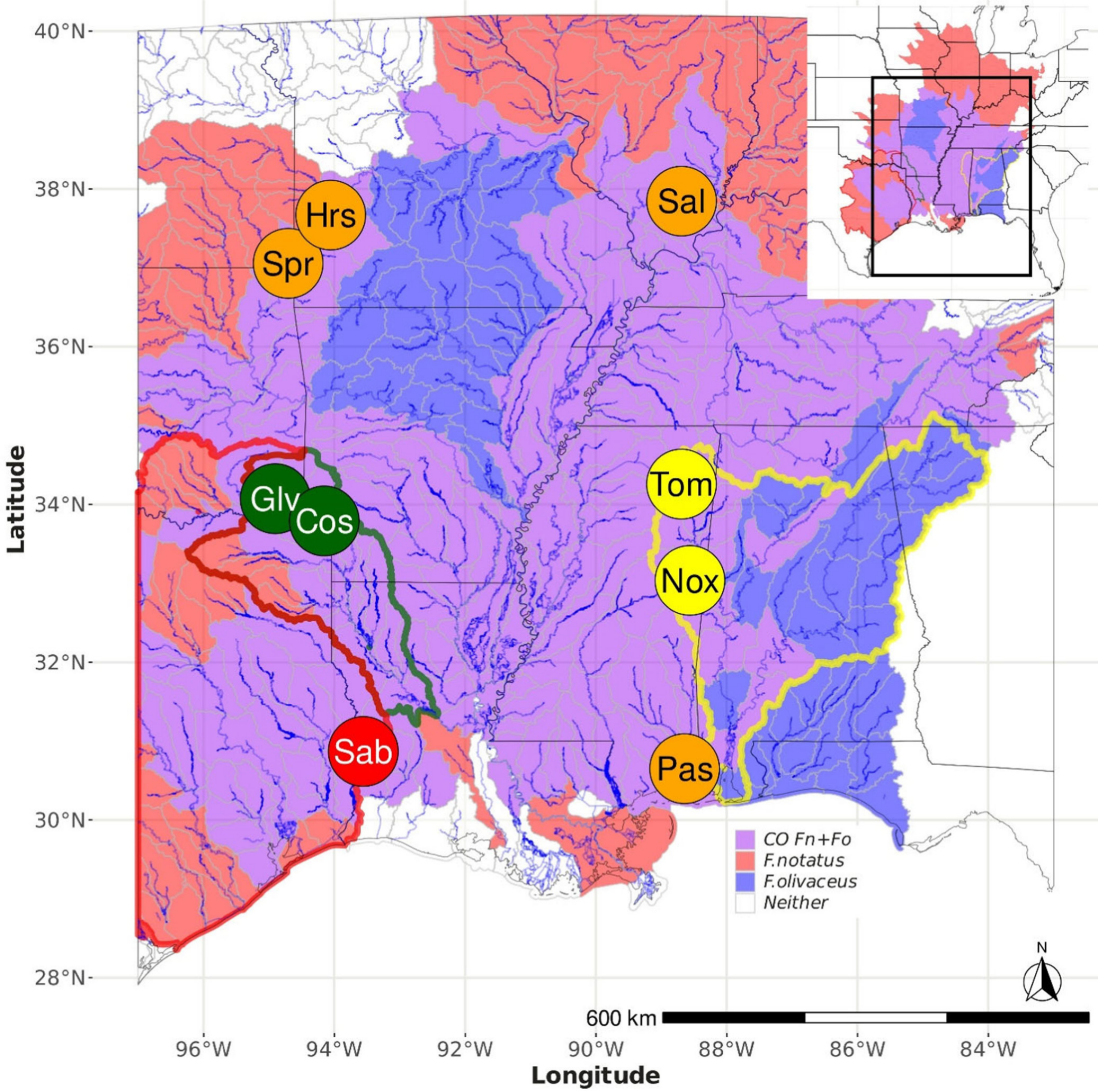
Species distributions of *Fundulus notatus* (red shading), *F. olivaceus* (blue shading) and their co‐occurrence (purple shading). Drainage areas (HUC8) are color coded according to presence of the respective species, and do not reflect relative abundance or finer within‐drainage species distributions. Contact zone sample sites are labeled according to Table 1. The geographic distributions of *F. notatus* clades are outlined, and contact zone sites are color coded according to phylogeographic clade to indicate the Western Gulf Slope (red outline and circle), the Red River basin (green outline and circles), the Mobile River basin (yellow outline and circles), and the coastal and Mississippi basin (orange circles).

We sampled nine contact zones between *F. olivaceus* and *F. notatus* throughout their geographic range (Figure 1, Table 1). Contact zones were selected based on the known phylogeography of *F. notatus*, since *F. olivaceus* populations do not exhibit strong phylogeographic structure. The Sabine River (Sab) was selected to represent the Western Gulf Slope clade. The Glover (Glv) and Cossatot (Cos) rivers in the southwestern Ouachita Highlands were selected to represent the Red River basin clade. The Tombigbee (Tom) and Noxubee (Nox) rivers represent the Mobile River basin clade. Finally, the Mississippi River basin clade was represented by contact zones in the Spring River (Spr), Horse Creek (Hrs), Saline River (Sal), and Pascagoula River (Pas). In each sampling region, the distribution of parental species and the center of the contact zone were known from earlier surveys (Duvernell & Schaefer, 2014; Schaefer et al., 2011, 2016; Steffensmeier et al., 2019). Within contact zones, sampling was restricted to the known region of co‐occurrence to ensure effective sampling of both species and possible hybrids. Individuals were sampled with a dipnet, and fin clips were preserved in 95% ethanol. Genomic DNA was extracted using the Qiagen DNeasy Blood and Tissue Kit (Qiagen). Samples from allopatric reference sites for each species (Table 1) were previously reported (Duvernell et al., 2019).

**TABLE 1:** Sample collection sites and sample sizes.

Sample	Coordinates	*n*
Contact zones		
Western Gulf Slope		
Sabine River (Sab)	30°52′9.84″ N, 93°33′28.44″ W	94
Red River basin		
Glover River (Glv)	34°4′22.08″ N, 94°54′14.04″ W	74
Cossatot River (Cos)	33°47′28.68″ N, 94°9′ 14.76″ W	82
Mississippi River basin		
Spring River (Spr)	37°3′21.6″ N, 94°42′18.72″ W	111
Horse Creek (Hrs)	37°40′5.52″ N, 94°3′22.68″ W	102
Saline River (Sal)	37°50′40.2″ N, 88°41′ 24.72″ W	75
Pascagoula River (Pas)	30°39′3.96″ N, 88°38′39.84″ W	95
Mobile River basin		
Tombigbee River (Tom)	34°15′26.64″ N, 88°41′30.84″ W	134
Noxubee River (Nox)	33°2′14.28″ N, 88°33′45.36″ W	98
Reference sites—*F. notatus*		
Colorado River	29°44′49.2″ N, 96°33′7.2″ W	11
Angelina River	31°35′7.8″ N, 94°49′45.48″ W	9
Twelve‐Mile Bayou	32°38′44.16″ N, 93°52′38.64″ W	12
Little River	33°37′14.88″ N, 93°51′40.32″ W	94
Cahokia Creek	38°53′41.28″ N, 89°55′18.48″ W	10
Chotard Lake	32°33′58.68″ N, 91°3′46.44″ W	11
Big Blue Hole	31°34′56.28″ N, 91°28′56.28″ W	18
Reference sites—*F. olivaceus*		
Old River Bayou	31°41′51″ N, 93°4′30.36″ W	13
North Fork	36°58′37.92″ N, 92°10′0.48″ W	10
Gasconade River	37°56′5.64″ N, 91°58′39.36″ W	5
St. Francis River	37°40′51.96″ N, 90°24′50.4″ W	10
Yellow River	30°42′16.92″ N, 86°52′53.04″ W	6

We employed GBS to obtain a reduced‐complexity genome representation of SNP genotypes following Elshire et al. (2011) as described above. This study included a combination of samples collected and reported for the first time in this study, as well as some samples that were reported in previous studies (Duvernell et al., 2019; Schaefer et al., 2016). All new samples were sequenced on the NovaSeq 6000 platform with 150 bp paired‐end sequencing. Previously reported samples, which included some samples from two of the contact zones (Glover and Cossatot; Schaefer et al., 2016) and all of the reference populations (Duvernell et al., 2019), were previously sequenced on a HiSeq instrument with single‐end sequencing.

All sequence reads were aligned to the linkage mapped and assembled *F. olivaceus* scaffolds using Bowtie 2 v.2.4 (Langmead & Salzberg, 2012). A single SNP library was generated for all reference and contact zone samples from reference aligned BAM files using the gstacks and populations programs in the Stacks 2 pipeline (Catchen et al., 2013; Rochette et al., 2019). Individuals that did not achieve a genotype quality (GQ) score of at least 30 across at least 50% of loci were resequenced, or the samples were discarded. We also filtered and removed SNP loci genotyped in fewer than 80% of all individuals in order to eliminate systematic differences among samples that could be introduced by differences in the sequencing platforms used.

### 3.3 Reconstructing historical relationships among populations

We reconstructed the historical relationships among populations and between species in contact zones and reference sites using the maximum likelihood approach implemented in TreeMix v. 1.13 (Pickrell & Pritchard, 2012). This model employs a graph‐based representation of population relationships to construct population and species relationships and infer gene flow events. We used a window size (‐k) of 100 and evaluated a number of migration edges (‐m) between 0 and 10. Individuals of hybrid ancestry were excluded from the TreeMix analysis by selecting only individuals with proportion of ancestry (*q*) > 0.95 for one species or the other from our Entropy analysis (see next section). We selected a set of phylogeographically informative loci from our Stacks SNP library by specifying a minimum allele frequency of 0.05, and minimum interlocus distance of 50,000 bp. A small subset of around 6% of loci exhibited substantial excess observed heterozygosity that may be caused by paralog alignment (Drury et al., 2016; Nunez et al., 2015). We removed all loci with >50% heterozygosity across all samples to remove possible sequence alignment artifacts.

### 3.4 Estimating admixture proportions of individuals

We estimated the proportion of ancestry (*q*) and interspecific heterozygosity (*Q*_12_) for individuals in each contact zone using the hierarchical Bayesian model implemented in Entropy (Mandeville et al., 2015; Shastry et al., 2021). Each contact zone was analyzed separately in Entropy with the number of populations, *k*, set to 2. Posterior distributions of parameters were estimated after merging three independent runs using Markov Chain Monte Carlo (MCMC) with 50,000 iterations sampling every 10th iteration after discarding an initial burn‐in of 2000 iterations. Population statistics were averaged over three replicate runs after convergence among runs was confirmed visually.

### 3.5 Genomic cline analysis

We used the Bayesian genomic cline (BGC) model (Gompert & Buerkle, 2011, 2012) to quantify variability in patterns of introgression among loci, chromosomes, and replicate contact zones. Bayesian genomic cline uses a hierarchical model to estimate cline parameters (*α* and *β*) describing introgression of each locus. The cline parameter *α* indicates a bias in the directionality of introgression relative to the genome average. Specifically, *α* indicates the magnitude and directionality of introgression at a single locus relative to the genome‐wide average. The cline parameter *β* specifies the rate of transition from one parental to the other. Negative values of *β* correspond to loci that introgress more readily (wider cline) than the genome‐wide average, and positive values correspond to loci that resist introgression (steeper cline). We used the BGC model to test the prediction that SNP markers mapped to Rb fusions in *F. notatus* would exhibit more positive *β* values than SNPs mapped to unfused linkage groups.

The BGC model requires specification of reference population samples to define population gene pools. Our first efforts to employ the BGC model utilized the same set of phylogeographically informative SNP loci as the TreeMix analysis, while using either individuals from our allopatric samples from neighboring drainages, or individuals from within contacts zones exhibiting Entropy *q*‐scores >0.95 as reference samples for each contact zone. However, numbers of available reference individuals from both sources, and the distributions of *q*‐scores varied by contact zone (dependent on prevalence of hybridization), making comparisons of BGC runs among contact zones problematic. Consequently, to simplify the analysis, we chose to use a set of “species‐diagnostic fixed loci” (between *F. olivaceus* and *F. notatus*) that could be used to estimate BGC parameters at the same set of loci across all contact zones.

Species diagnostic fixed loci were selected from the Stacks SNP library by constructing a geographically diverse set of *F. notatus* and *F. olivaceus* reference individuals in equal proportions (see Supporting Information) and applying a minimum allele frequency of 0.48, and maximum heterozygosity of 0.02. The resulting loci were then extracted from the SNP library for all contact zones. We confirmed that the fixed loci provided coverage across all 24 *F. olivaceus* scaffolds (Figure S1). We used Principle Coordinates Analysis (PCoA, adegenet package) of Euclidean distances to visualize relationships among samples to confirm that phylogeographic variation was effectively removed from our set of fixed loci when compared to phylogeographically informative loci (Figure S2).

Bayesian genomic cline analyses of fixed loci included three independent MCMC chains with 100,000 iterations sampled every tenth iteration following an initial burn‐in of 50,000. Output of the three runs was combined after determining convergence on the same stationary distributions using ClinePlotR (Martin et al., 2020). Bayesian genomic cline input files were constructed from VCF files using ClineHelpR (Martin et al., 2021). Bayesian genomic cline loci were considered outliers if either the 95% credibility intervals for *α* or *β* did not overlap zero, or the median of the posterior distribution exceeded the probability distribution's quantile interval (Gompert & Buerkle, 2011).

We used the inbreeding coefficient, *F*_IS_, generated from species diagnostic fixed loci, as a summary estimator of nonrandom mating between species within contact zones. The inbreeding coefficient is the reduction in heterozygosity of an individual due to nonrandom mating (Hartl & Clarck, 2006). The use of loci that exhibit fixed differences between the species effectively removed the influence of intraspecies population dynamics. In a contact zone in which only parental genotypes are present (no hybrids), all individuals would be homozygous at species diagnostic loci, and *F*_IS_ would equal one. Alternatively, if *F*_IS_ was equal to zero, this would indicate that assumptions of Hardy–Weinberg were approximately met. This would include nondiscriminant mating between species, and some level of hybrid offspring viability. Interspecific *F*_IS_ estimates were derived from summary statistics generated by the populations program in Stacks.

## 4 RESULTS

### 4.1 Reference genome scaffold assemblies and identification of linkage group fusions

Contigs in the published unmapped draft genome assembly for *F. olivaceus* (Johnson et al., 2019) were mapped to scaffolds using an F2 family of 65 *F. olivaceus* offspring. The *F. olivaceus* scaffolded assembly utilized 1839 unique map‐informative SNP markers to construct 24 linkage groups totaling 825 Mb (67.6%) of the published *F. olivaceus* draft genome. Chromosome fusions in the *F. notatus* genome were identified with a separate linkage map constructed from an F2 family of 53 *F. notatus* offspring. The *F. notatus* map included 5860 markers in 20 linkage groups totaling 916 Mb (75.1%) of the published draft genome. Alignment of the assembled genomes identified the four paired linkage groups that have undergone fusion in an *F. notatus* ancestor. These four paired linkage groups were numbered (1,6), (9,15), (10,19), and (14,20) in our *F. olivaceus* linkage map.

### 4.2 Population phylogeography

After quality filtering, there were a total of 453,154,662 reads from 1269 individuals distributed over nine contact zones and 12 reference populations (7 *F. notatus* and 5 *F. olivaceus*). After quality filtering of SNPs, the median read coverage was 26x, and the median proportion of loci with GQ > 30 was 82%.

**FIGURE 2:**
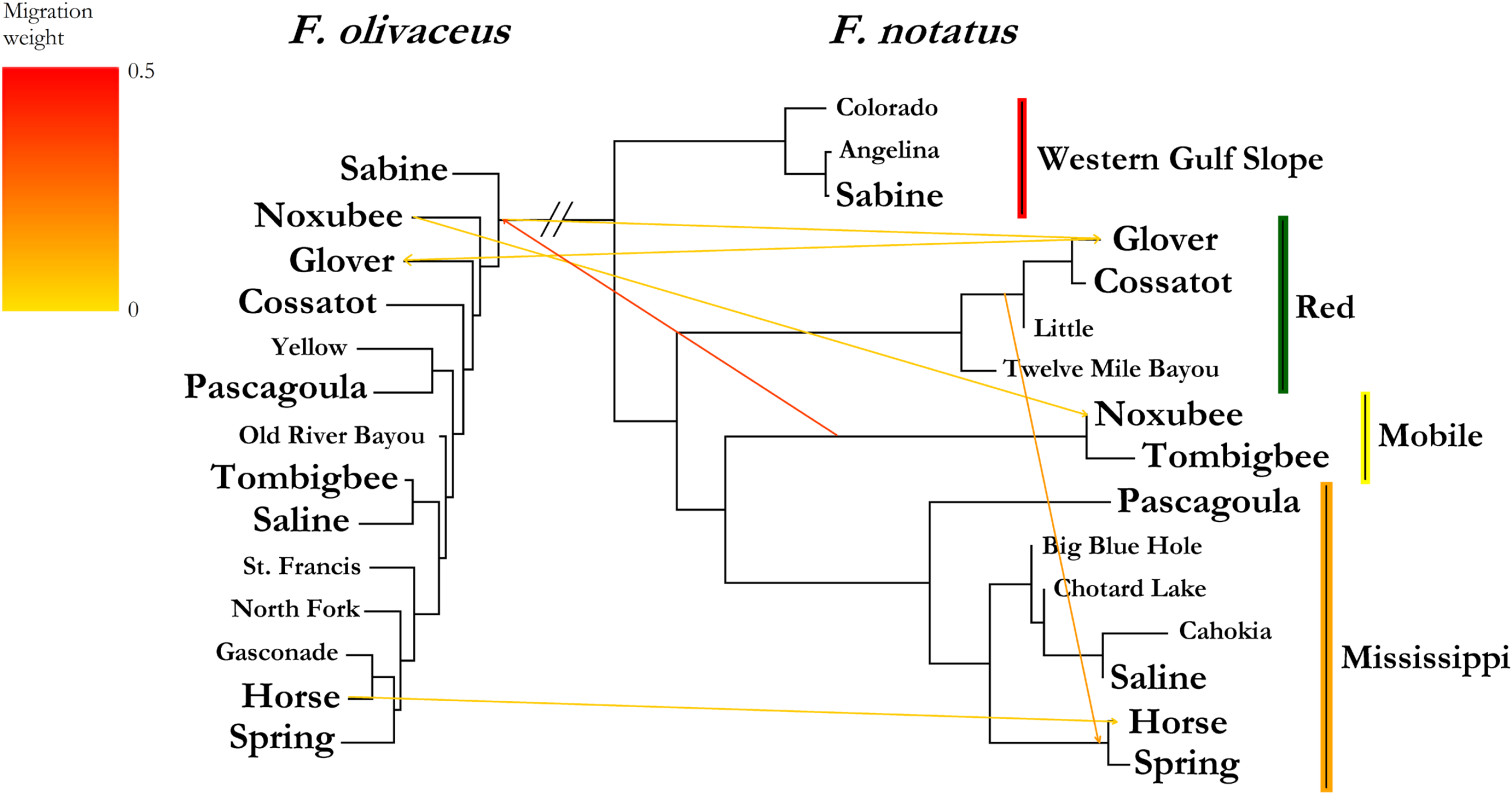
Maximum likelihood phylogeny of contact zone and reference samples of *Fundulus olivaceus* and *F. notatus*. Inferred migration events are indicated by arrows color‐coded according to their weight. Contact zone populations are labeled in bold, and *F. notatus* phylogeographic clades are labeled on the right.

A maximum likelihood phylogeny constructed with TreeMix using 3314 phylogeographically informative loci confirmed the relationships among *F. notatus* clades (Figure 2). The phylogeny included all allopatric reference samples, as well as populations of each species at all nine contact zones. This phylogeny supported the phylogeographic structure of *F. notatus* into four broadly supported and previously reported clades, and comparative lack of phylogeographic structure in *F. olivaceus* (Duvernell et al., 2013, 2019). Four of the first six migration edges inferred by TreeMix were interspecific, and connected branch tips of samples from contact zones (Horse, Glover, Noxubee). The inferred magnitude of <2% for these branch tip migration edges was consistent with our selection of contact zone individuals with *q* > 0.95, where some low‐level admixture (i.e., <5%) was possible in some of these active hybrid zone individuals. The fifth migration edge identified an intraspecific *F. notatus* migration event connecting the Red River drainage clade to the Horse/Spring clade with an admixture value of 10%. The most substantial migration edge inferred in the analysis connected the root of the *F. notatus* Mobile drainage clade to the root of the *F. olivaceus* clade with an admixture value estimated at 36%.

### 4.3 Contact zone analysis

**FIGURE 3:**
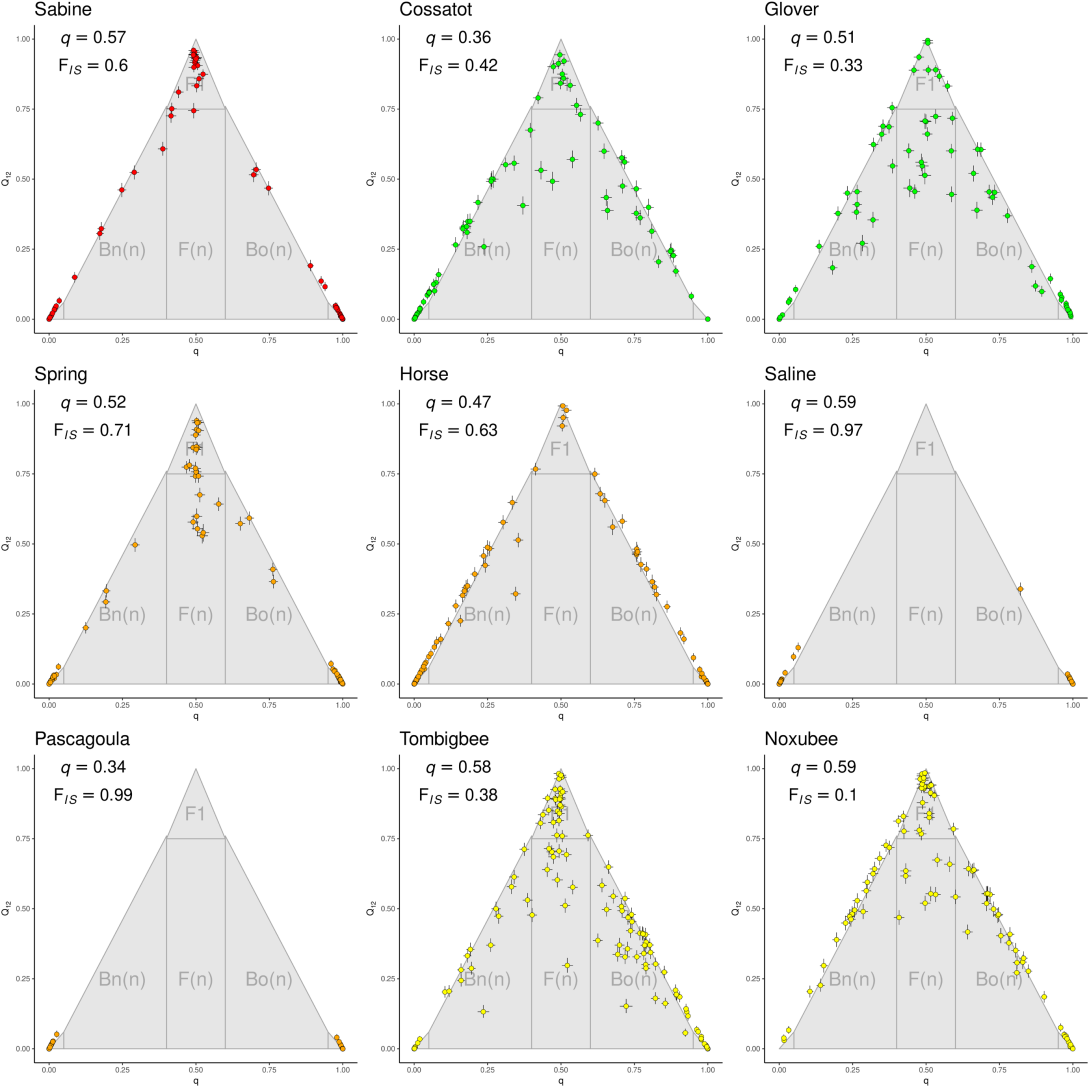
Proportion of ancestry (*q*) versus interspecific heterozygosity (*Q*_12_) for each individual within each contact zone from species‐diagnostic fixed SNP loci. In each plot, pure *Fundulus notatus* appear in the lower left and *F. olivaceus* in the lower right corners. F1 hybrids appear at the apex of each triangle plot. Multigeneration hybrids (e.g., F2, F3) occupy the middle space while multiple backcross generations (Bn(*n*), Bo(*n*)) occur along the left and right margins of the triangles. The mean species ancestry, and mean *F*_IS_ for each site is indicated next to each triangle plot. Colors correspond to phylogeographic clades (Figure 1).

**FIGURE 4:**
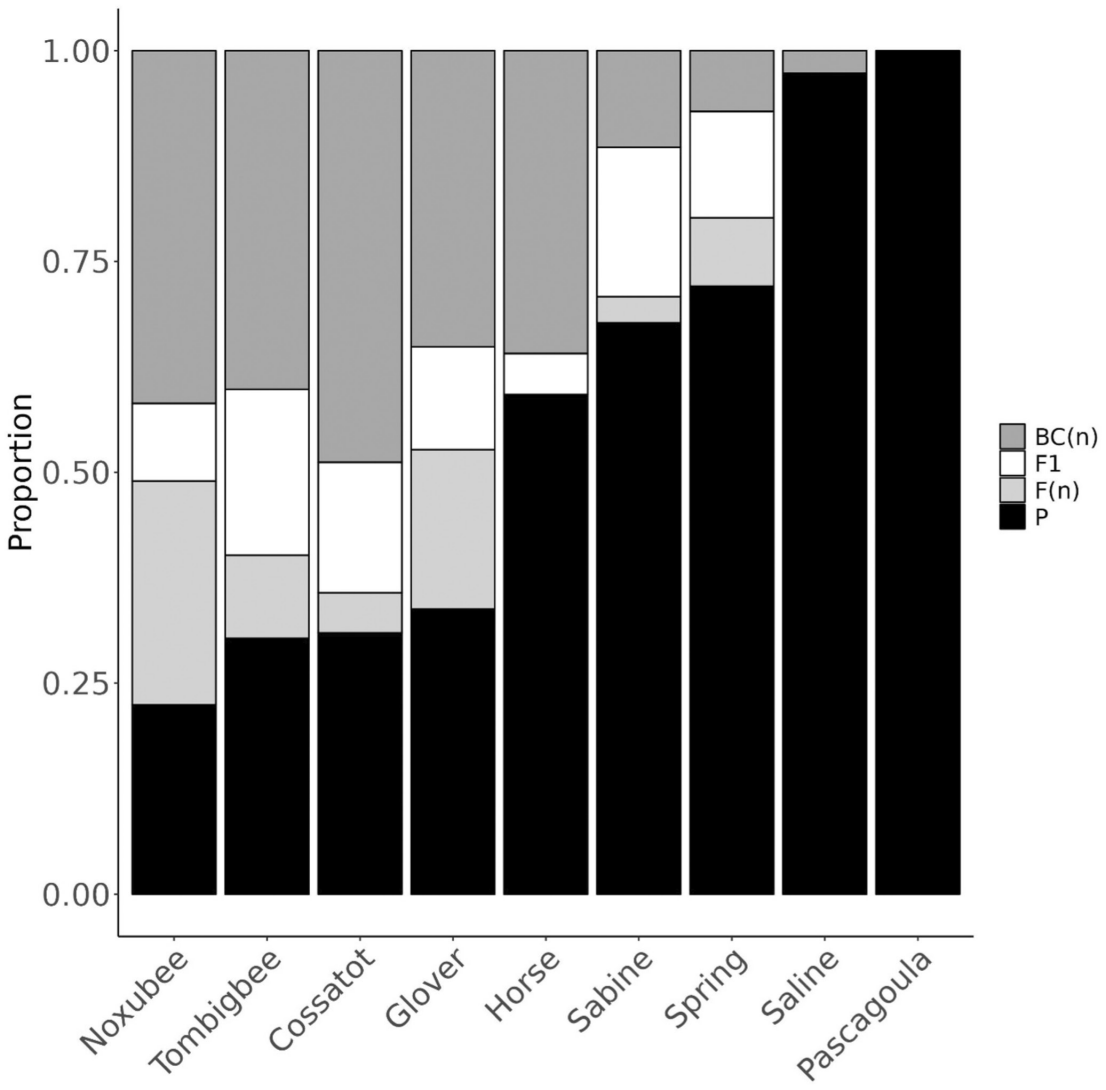
Inference of proportions of hybrid classes from proportion of ancestry (*q*) and interspecific heterozygosity (*Q*_12_) from Entropy analysis (this figure). We classified individuals as likely F1 (*q* 0.4–0.6; *Q*_12_ > 0.75), F(*n*) (*q* 0.4–0.6, *Q*_12_ 0.25–0.75), back‐cross (BC(*n*)) (*q* 0.05–0.4 or *q* 0.6–0.95), or parental (P) (*q* < 0.05 or *q* > 0.95).

We sampled similar proportions of both species in all nine contact zones. The overall average proportion of *F. olivaceus* was 0.36 in the Cossatot and 0.34 in the Pascagoula; it ranged between 0.45 and 0.60 in all other contact zones. We observed substantial variation in levels of hybridization among contact zones. There was also variation in distribution of ancestry proportion (*q*) and interspecific heterozygosity (*Q*_12_) among contact zones (Figure 3). At one extreme, hybridization was virtually absent in the Pascagoula and Saline rivers. In the Horse Creek and Sabine River contact zones, extensive backcrossing was evident in both species, but there were comparatively few F2 individuals (Figure 4). In contrast, the Spring River exhibited relatively high proportions of putative F1 and F2 individuals, and fewer backcross individuals. Hybridization was most extensive in the Cossatot, Glover, Tombigbee, and Noxubee rivers where multigeneration hybrid and backcross hybrid individuals were prevalent. Backcross hybridization appeared symmetrical, with similar numbers of backcross hybrids for each species, in every contact zone (Figure 3).

### 4.4 Alpha and beta distributions by contact zone and by chromosome

For BGC analysis, we identified 2236 species‐diagnostic fixed loci, 1767 (79%) of which mapped to the 24 *F. olivaceus* scaffolds. There were 595 fixed loci shared between the randomly selected and species‐diagnostic loci. That is, 34% of mapped, species‐diagnostic loci were present among the randomly selected loci, and 18% of the randomly selected loci were included among the species diagnostic loci.

**FIGURE 5:**
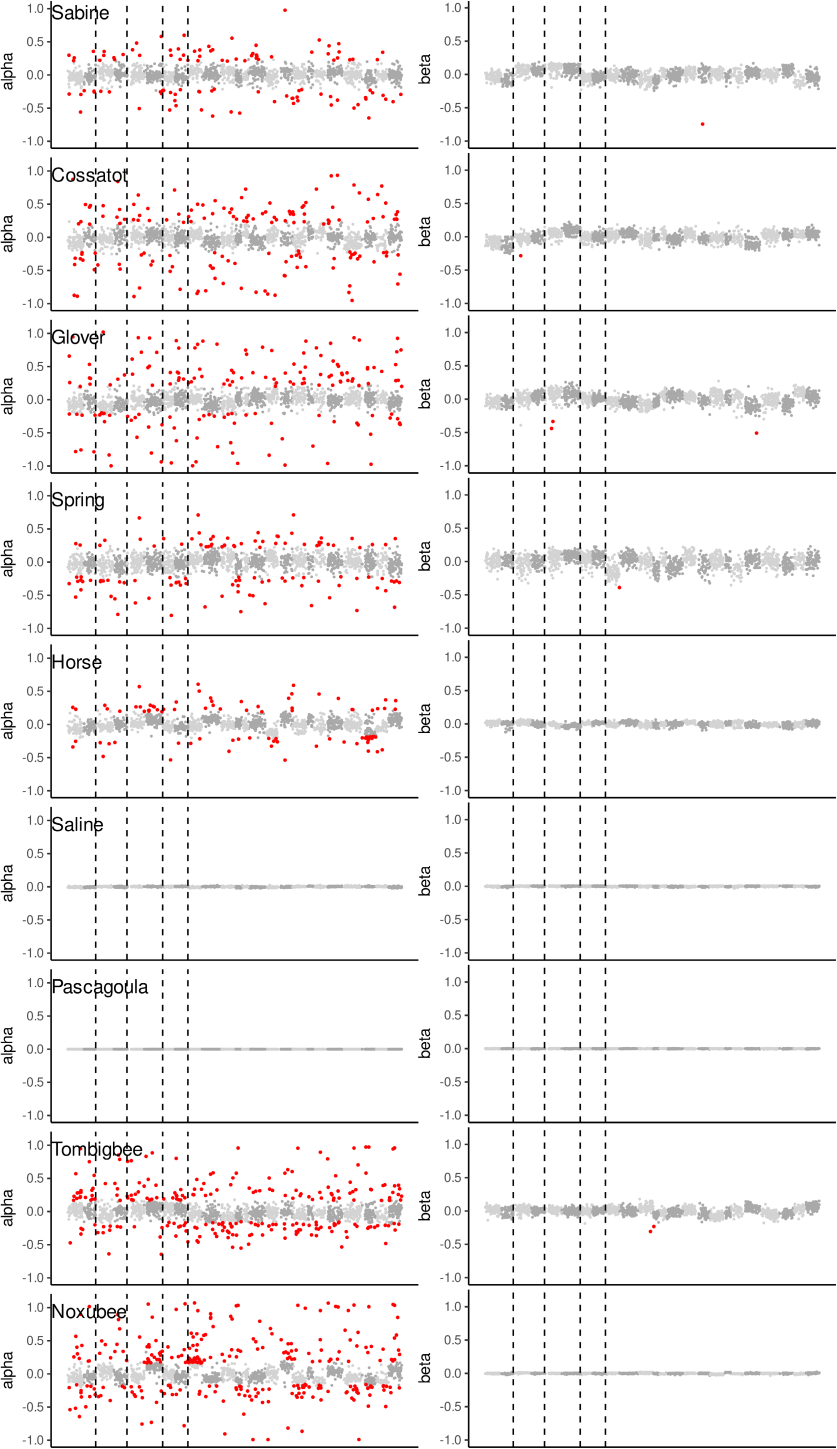
Distribution of genomic cline parameters of species diagnostic SNPs along each linkage group. Linkage groups are alternatingly indicated by light and dark gray symbols. Vertical lines separating the first four pairs of linkage groups identify fusions in *F. notatus* linkage groups relative to *F. olivaceus*. (a) Excess of ancestry (*α*) in *F. notatus* (neg) or *F. olivaceus* (pos) relative to genome‐wide average. (b) Rate of transition in ancestry (*β*). Outlier SNPs are marked in red.

Genomic cline parameters *α* and *β* were highly variable in some contact zones, and invariant in others (Figure 5). There was no evidence of consistent differences in patterns of *α* or *β* statistics between inferred fused and unfused linkage groups in any of the contact zones. The proportion of loci with *α* that differed significantly from the genome‐wide average (*α* outliers) ranged from none in the Pascagoula and Saline contact zones to as high as 14% and 16% of loci in the Tombigbee and Noxubee contact zones, respectively (Figure 5; Table S1). The proportions of outlier loci that were positive or negative (i.e., biased in favor of one species or the other) were similar within each contact zone, and outliers were distributed among all linkage groups. Similarly, the mean of all *α* was very close to zero in all contact zones, and across all linkage groups (Table S1).

As with *α*, per locus estimates of *β* exhibited higher variance in some contact zones than in others (Figure 5). The mean and variance in *β* were both near zero in the Pascagoula and Saline contact zones, where hybridization was limited. Variance in *β* was highest in the Sabine, Cossatot, Glover, and Spring contact zones where hybridization rates were high. Across all contact zones, there were only nine negative *β* outliers and no positive outliers (Figure 5; Table S1). The average value of *β* did not differ between fused and unfused linkage groups.

### 4.5 Comparison of hybridization rates across contact zones

**FIGURE 6:**
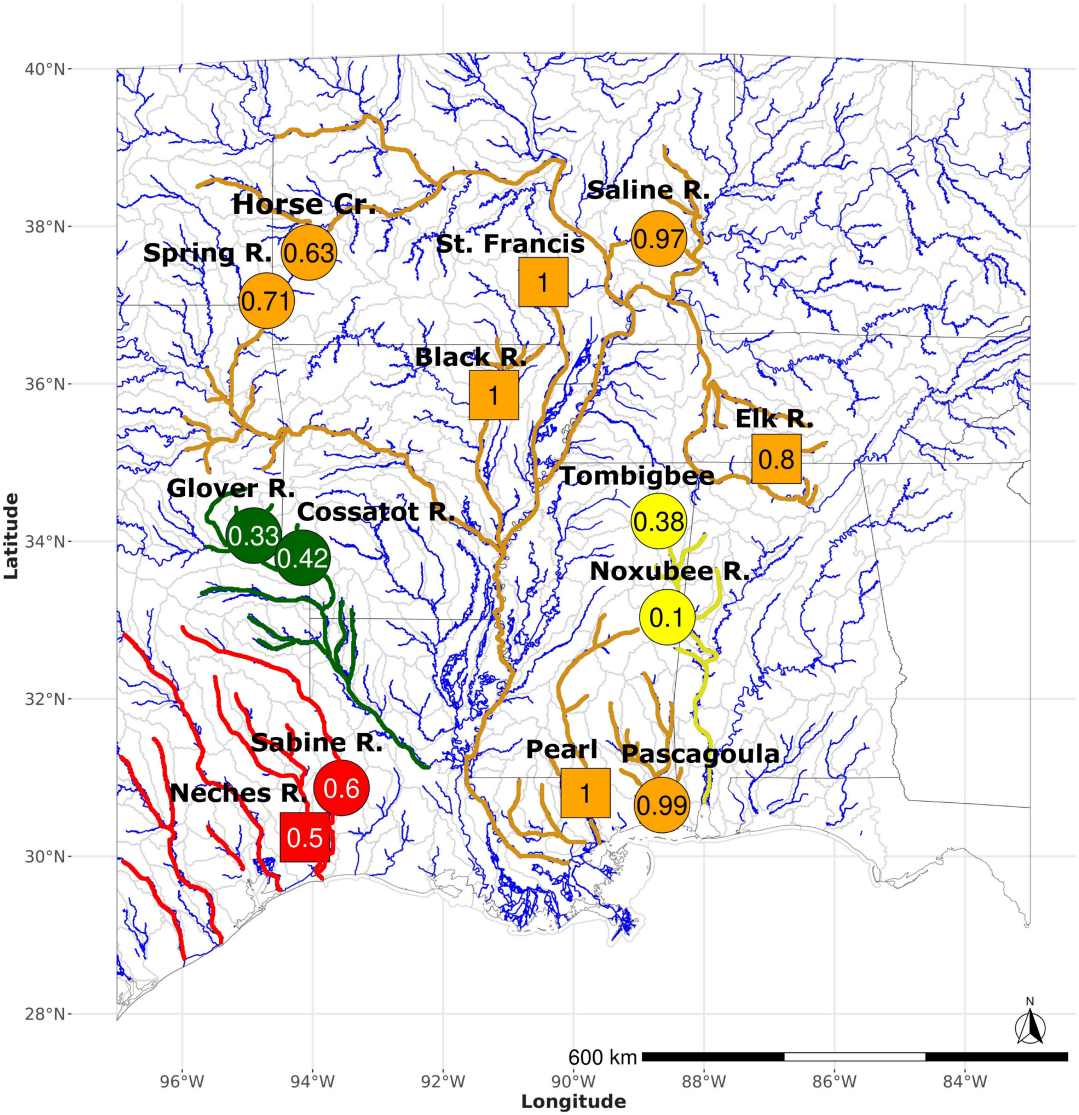
Geographic distribution of hybridization rates inferred from estimates of mean *F*_IS_ at species‐diagnostic SNP loci. Sites are color coded based on *F. notatus* phylogenetic clade (orange—Mississippi clade, yellow—Mobile clade, green—Red River clade, red—Western Gulf Slope clade). Sites with estimates from this study are indicated with circles and estimates from previous studies (Duvernell & Schaefer, 2014; Steffensmeier et al., 2019) are indicated with squares. Samples with *F*_IS_ = 1 exhibited individuals of both species, and no heterozygous genotypes.

Genome‐averaged per locus *F*_IS_ estimates at species‐diagnostic fixed loci provided a summary of overall extent of hybridization for comparison among contact zones. We compared genome‐averaged *F*_IS_ estimates at fixed loci in this study to multilocus *F*_IS_ estimates based on a small number of targeted species‐diagnostic PCR‐RFLPs in previous studies (Table 2; Duvernell & Schaefer, 2014; Steffensmeier et al., 2019). Among the six contact zones in this study for which *F*_IS_ values have been previously reported (Table 2), there was a strong correlation (*r* = .88, *p* = .02) supporting the validity of the cross‐study comparisons using different sets of SNP markers. Altogether, estimates of *F*_IS_ were available for 14 contact zones distributed over most of the co‐occurrence of the two species (Figure 6).

**TABLE 2:** Summary of contact zones and interspecific inbreeding coefficients.

Region drainage	*F*_IS_ ^a^	*F*_IS_ ^b^
Western Gulf Slope		
Sabine River	0.60	0.41
Neches River		0.50
Red River basin		
Glover River	0.33	0.22
Cossatot River	0.42	
Mobile River basin		
Tombigbee River	0.38	0.02
Noxubee River	0.10	
Mississippi River basin		
Spring River	0.71	0.16
Horse Creek	0.63	
Saline River	0.97	0.79
Pascagoula River	0.99	0.92
Black River		1.0
Elk River		0.80
Pearl River		1.0
St. Francis River		1.0^c^

^a^ Calculated using 2236 species diagnostic loci in this study.

^b^ Calculated using five nuclear PCR‐RFLP loci (Duvernell & Schaefer, 2014).

^c^ Calculated using one nuclear PCR‐RFLP locus (Steffensmeier et al., 2019).

Sites where hybridization rates were very low or absent (*F*_IS_ ~ 1) were all restricted to contact zones in the Mississippi River basin and adjacent coastal drainages. These included the Pascagoula and Pearl coastal drainages, as well as the Saline, Elk, Black, and St. Francis River drainages in the Mississippi River basin. This study indicated that hybridization was entirely absent in the Pascagoula River and nearly so in the Saline River. Previous studies of other contact zones utilizing a small number of species‐diagnostic PCR‐RFLP loci detected an absence of heterozygous genotypes among mixed‐species samples collected in the Pearl, Black, and St. Francis Rivers, and only minimal hybridization in the Elk River (Duvernell & Schaefer, 2014; Steffensmeier et al., 2019). The *F. notatus* populations in all of these drainages belong to the Mississippi basin clade (Figure 2; Duvernell et al., 2019). Hybridization rates were moderately elevated in the northwest portion of the co‐occurrence range, in the Spring River and Horse Creek contact zones. While the *F. notatus* populations in both of these drainages belong to the Mississippi clade, their gene pools are also approximately 10% admixed with the Red River clade (Figure 2; Duvernell et al., 2019). Contact zones in the Mobile basin (Tombigbee, Noxubee), Red River basin (Glover and Cossatot), and Western Gulf Slope (Sabine and Neches) all exhibited reduced *F*_IS_ and higher hybridization rates. We found that population phylogeographic history, and specifically *F. notatus* phylogeographic history, was a strong predictor of hybridization rates. Significant differences in *F*_IS_ existed among drainage systems partitioned based on the four *F. notatus* clades overall (Kruskal–Wallis *χ* ^2^ = 10.4, df = 3, *p* = .015).

## 5 DISCUSSION

The topminnows *F. olivaceus* and *F. notatus*, with broad, extensively overlapping geographic ranges, provide an opportunity to study mechanisms of reproductive isolation that promote and maintain species diversity. The most striking observation in our study was the breadth of contrasting hybridization rates and patterns exhibited across isolated drainages. In some contact zones, we observed a virtual hybrid swarm, with a prevalence of hybrid classes (F1, F2, multigeneration backcrosses), and low interspecific *F*_IS_ consistent with close to random mating and at least partial hybrid viability. In other contact zones, both species were observed co‐occurring within the same habitats in equal proportions, with no F1 or early generation backcross individuals, and correspondingly, *F*_IS_ nearly 1. Our results, based on a genome‐wide distribution of SNPs on all linkage groups, confirmed previous reports of similarly wide ranging hybridization rates, which were based on a small number of loci (Duvernell & Schaefer, 2014; Schaefer et al., 2011).

Contrasting patterns of hybridization in this study suggest that reproductive isolation is highly variable among populations across drainages. The genetic basis of reproductive isolation is supported by previous work. A study of mate selection (probability of spawning) and backcross hybrid offspring viability (hatching success) reported evidence of both prezygotic (conspecific mate preference) and postzygotic (low hatching success) barriers (Vigueira et al., 2008). The parents included in that study were from drainages in which hybridization is virtually absent (Pearl and Pascagoula rivers; as reported in Duvernell & Schaefer, 2014 and in this study). In common garden hybrid zone trials conducted with *F. notatus* and *F. olivaceus* parents placed in stream mesocosms, hybridization rates in the mesocosms for the Tombigbee and Pascagoula populations (J. F. Schaefer, unpublished data) matched high and low hybridization rates, respectively, observed in natural contact zones in this study.

Variability in rates of hybridization could also be attributable to ecological factors that vary among drainages and contact zones, or due to environmental degredation, as environmental factors may account for variation in mate recognition and breakdown of prezygotic isolation (Seehausen et al., 2008; Ward & Blum, 2012). However, previous studies have indicated hybridization rates may be at best only weakly associated with drainage‐level environmental variables (Duvernell et al., 2007; Duvernell & Schaefer, 2014; Schaefer et al., 2011, 2016), and do not seem to be associated with observed habitat disturbance levels (Duvernell & Schaefer, 2014).

### 5.1 Is reproductive isolation localized to specific chromosomes?

We set out to evaluate whether introgression patterns varied throughout the genome in a consistent pattern among contact zones, and to test the hypothesis that chromosomal differences resulting from Rb fusions contributed to reproductive isolation in hybridizing populations. A limitation of this approach was that the complete absence of hybrids in drainages inferred to have the strongest reproductive isolation precluded those contact zones (most notably Pascagoula and Saline) from providing informative genomic cline data.

We sought to test whether the SNP markers on the four pairs of fused linkage groups inferred in the *F. notatus* genome would exhibit steeper genomic clines than the 16 unfused linkage groups. Our results did not uncover any consistent patterns among linkage groups for genomic clines based on either *α* or *β* parameters or support a specific role of Rb fusions in promoting reproductive isolation. We detected no positive *β* outliers that would be indicative of reproductive barriers in any linkage groups, and there were no consistent differences in inferred chromosome‐level mean *β* across independent contact zones. We found no evidence of consistent differences in genomic clines between fused and unfused linkage groups, respectively. This study fits with some other studies and systems in which Rb fusions have appeared to not limit gene flow per se or disproportionately contribute to reproductive isolation between species that differ in karyotype (Horn et al., 2012; Potter et al., 2015, 2017). Our results seem to contrast with a population study of a pair of closely related killifishes in the genus *Lucania* that indicated a single Rb fusion is associated with behavioral reproductive isolation (Berdan et al., 2014, 2021).

We found that substantial numbers of *α* outliers were indicated in contact zones where hybridization was extensive. Consistent directional patterns of the *α* parameter could indicate biased directionality of introgression between species for some genomic regions relative to the genome as a whole. However, both positive and negative outliers (directionality favoring one species or the other) were generally distributed uniformly across linkage groups, and in similar proportions (Figure 5). There were no consistencies in *α* outliers among contact zones, with no consistent patterns in *α* overall emerging among linkage groups or contact zones. The proportion of loci that were identified as *α* outliers was strongly inversely correlated with *F*_IS_ (*r* = −.96). In the absence of any consistent patterns of *α* outliers among linkage groups, we interpret these results as uninformative overdispersion of the *α* parameter.

It remains unclear the nature and extent of reproductive isolation in topminnows. It is possible that Rb fusions were not found to be disproportionately associated with steeper genomic clines if, perhaps, postzygotic isolation is distributed more widely across chromosomes, and not inherently associated with specific linkage groups or Rb fusions. These topminnow species are estimated to be of Pliocene origin, having diverged over several million years (Duvernell et al., 2019). In species that have been reproductively isolated for a substantial period of time, genome incompatibilities are predicted to occur throughout the genome, possibly obscuring initial speciation genes (Faria & Navarro, 2010; Navarro & Barton, 2003). The results of this study indicate that reproductive barriers are not localized to specific linkage groups or fused chromosomes, at least in drainages where the prevalence of hybridization provided an assessment.

### 5.2 A phylogeographic explanation for reproductive isolation

Variable hybridization rates may result from diverse histories of sympatry among populations, even between reciprocally monophyletic species (Zieliński et al., 2019). Our combined assessment of hybrid zone studies of these species suggested that there may be a strong phylogeographic explanation for reproductive isolation. We found that hybridization was most limited or absent from contact zones in drainages distributed along a north–south region that spanned the center of both species' ranges (Figure 6). These drainages are distributed over multiple ecoregions (i.e., Gulf Coastal Plain, Mississippi Alluvial Plain, Ozark Highland, Interior Plain) in drainages of varying anthropogenic modification, but all are tributaries in the Mississippi River basin, or coastal drainages that shared a connection to the Mississippi River as recently as the late Pleistocene (Galloway et al., 2011). Correspondingly, the *F. notatus* populations in these drainages all belong to the Mississippi basin phylogenetic clade (Figure 2; Duvernell et al., 2019; Duvernell & Schaefer, 2014). Both species are inferred to have experienced late Pleistocene northward range expansions. The Mississippi clade of *F. notatus* expanded its range along the Mississippi River dispersal corridor into much of its present day geographic distribution, and *F. olivaceus* did much the same, as well as expanding its range into coastal drainages where the other three *F. notatus* clades (Western Gulf, Red River, and Mobile basin) are distributed (Duvernell et al., 2019).

The phylogeographic histories of *F. notatus* and *F. olivaceus* and the geographic variation in hybridization rates lead to our interpretation that hybridization rates, and the inferred history of contacts among extant lineages are connected. Hybridization rate is a population‐level trait, and the underlying variation for prezygotic reproductive isolation could be segregating at different frequencies among populations (Cutter, 2012). It could also have evolved independently in different regions of the species' distributions, possibly driven by reinforcement selection processes (Kozak et al., 2015; Moran et al., 2018; Noor, 1999; Servedio & Noor, 2003), which could vary along phylogeographic divisions among populations of one or both species. The phylogeographic distribution of hybridization rates among contact zones supports a hypothesis that reproductive isolation evolved between *F. notatus* and *F. olivaceus* most completely within the lower Mississippi River basin and proximate coastal drainages. As both species underwent late Pleistocene range expansions, *F. olivaceus* populations came into secondary contact with new clades of *F. notatus* and may have experienced a breakdown of some genetic isolating mechanisms resulting in more extensive hybridization. This interpretation of the phylogeographic pattern of variation in hybridization rates suggests that the prevalence of reproductive isolation is a function of the age and history of sympatry between populations as well as the underlying genetic basis for reproductive isolation.

### 5.3 Possible implication of deep introgression for *F. notatus* karyotype evolution

The evolution of karyotypic variation among *F. notatus* and *F. olivaceus* populations is complicated by the presence of one karyotype in *F. olivaceus* (*n* = 24; Chen, 1971), and two karyotypes in *F. notatus* (*n* = 20, 22; Black & Howell, 1978; Chen, 1971). The ancestral condition of *n* = 24 has been inferred based on other members of the genus (Chen, 1971). Interestingly, the Mobile drainage clade of *F. notatus*, with a suggestively intermediate karyotype of *n* = 22, is not basal within the intraspecies phylogeny of *F. notatus* (Figure 2), raising the intriguing question of how the distinctive karyotype of the Mobile drainage clade evolved. This study did not include a genetic map of the Mobile drainage clade of *F. notatus* that could have revealed the homology of fused chromosomes in that drainage. However, this study did detect a substantial historical admixture exchange of 36% between the base of the *F. olivaceus* clade, and the base of the *F. notatus* Mobile clade. Given that the Mobile clade is not basal within the *F. notatus* phylogeny, this interspecific admixture event could offer insight into the history of chromosome evolution in the Mobile basin that would require more extensive genome reconstructions to investigate.

## 6 CONCLUSIONS

Analyses of contact zones between *F. notatus* and *F. olivaceus* demonstrated substantial variation in hybridization rates among populations and drainages. We assessed genomic clines to evaluate whether consistent patterns emerged in rates of introgression throughout the genomes, and tested a hypothesis that interspecific chromosomal differences, marked by multiple Rb fusions, contributed to reproductive isolation. We found that genomic clines were uniform throughout the genome and that there were no differences between fused and unfused linkage groups. The variation in hybridization rates among drainages suggests that reproductive isolation varies substantially among populations. A phylogeographic pattern in rates of hybridization suggests a possible role of phylogeographic history in determining reproductive isolation among populations of the respective species. There is still much to learn about the genetic basis of reproductive isolation between species of topminnows, and whether and how reproductive isolation varies between the species and geographically among populations of the respective species.

## AUTHOR CONTRIBUTIONS

**David D. Duvernell:** Conceptualization (equal); data curation (equal); formal analysis (lead); funding acquisition (equal); investigation (equal); methodology (equal); project administration (equal); resources (equal); supervision (lead); writing – original draft (lead); writing – review and editing (lead). **Naznin S. Remex:** Formal analysis (supporting); investigation (supporting); project administration (supporting). **Jeffrey T. Miller:** Formal analysis (supporting); methodology (supporting); software (supporting). **Jacob F. Schaefer:** Conceptualization (equal); data curation (equal); formal analysis (equal); funding acquisition (equal); investigation (equal); methodology (equal); project administration (equal); resources (equal); supervision (equal); writing – original draft (supporting); writing – review and editing (supporting).

## BENEFIT‐SHARING STATEMENT

Benefits Generated: Benefits from this research accrue from the sharing of our data and results on public databases as described above.

## Supporting information


Data S1

Table S1

## Data Availability

Genetic Data: All contact zone and reference sample fastq files are deposited in the SRA (BioProject PRJNA923285). Reference genome assembly files of *F. olivaceus* and *F. notatus* (FASTA format), and SNP data files (VCF format) are available for download in Scholars' Mine (https://scholarsmine.mst.edu/biosci_facwork/396). Sample Data: Metadata are also stored in the SRA (BioProject PRJNA923285).
