# Supplementary material for: Variable rates of hybridization among contact zones between a pair of topminnow species, Fundulus notatus and F. olivaceus
Source: Ecol Evol. 2023 Aug 8;13(8):e10399. doi: 10.1002/ece3.10399 (PMC10408002; doi:10.1002/ece3.10399)

**Supplementary Materials**

Construction of *F. olivaceus* and *F. notatus* linkage maps

We constructed F2 families of *F. olivaceus* and *F. notatus*, and genotyped F2 offspring using genotype by sequencing as described in the methods section.

We aligned short reads from the *F. olivaceus* F2 family to the *F. olivaceus* draft genome contigs (Johnson et al., 2019), and then assembled those contigs into *F. olivaceus* linkage groups as performed in (Miller et al., 2019). Reads were aligned and indexed with bowtie2, filtered for low-quality alignments with Samtools, and de-duplicated with SAMBLASTER (Faust & Hall, 2014; Langmead & Salzberg, 2012b; Li et al., 2009). We visualized the Samtools per site depth output in R to set the minimum and maximum coverage limits of SNP sites. We then used BEDTools to generate a bed file of intervals along the scaffolds that had sufficient coverage for genotype calling (Quinlan & Hall, 2010; R Core Team, n.d.). Genotypes were called on the alignments with *freebayes* and converted to bcf files with Bcftools (Danecek et al., 2021; Garrison, 2010/2018). We then used Bcftools to filter the SNPs from the dataset that fell outside the bed file coverage intervals. We then used PLINK to filter low frequency SNPs (likely sequencing error) and segregation errors within each mapping family (Purcell et al., 2007). The PLINK output was converted to the csvr format with the PLINKtoCSVR function adapted from PLINK2RQTL (Arends, 2014/2019). Genotypes for each family were loaded into rQTL with an ‘intercross’ design (Broman et al., 2003). Markers with more than 5 missing genotypes were dropped from the dataset. We formed the initial linkage groups by grouping *F. olivaceus* scaffolds that fall on the same chromosome when aligned to the mapped *Fundulus heteroclitus* genome. A small number of scaffolds that were linked to 3 or more *F. heteroclitus* chromosomes were dropped from the dataset. We then established linkage groups for each family by grouping markers with a minimum LOD score of 8 and recombination frequency of 0.15. To correct the phase of each linkage group, we visualized recombination frequency and linkage between the initial linkage groups. We then used the ‘switchAlleles’ function in rQTL to iteratively correct the phase of groups that were linked to one another and re-visualized recombination frequency and linkage to confirm the correction. Markers in each linkage group were then ordered with the traveling salesperson problem solver (Delaneau et al., 2017; Monroe et al., 2017). The mapping distance between markers was then estimated with the Kosambi mapping function with 100,000 iterations and an error probability of 0.04. This mapping process was repeated with the *F. notatus* F2 family to obtain an *F. notatus* map.

References:

Arends, D. (2019). DannyArends/PLINK2RQTL [R]. https://github.com/DannyArends/PLINK2RQTL (Original work published 2014)

Broman, K. W., Wu, H., Sen, Ś., & Churchill, G. A. (2003). R/qtl: QTL mapping in experimental crosses. Bioinformatics, 19(7), 889–890. https://doi.org/10.1093/bioinformatics/btg112

Danecek, P., Bonfield, J. K., Liddle, J., Marshall, J., Ohan, V., Pollard, M. O., Whitwham, A., Keane, T., McCarthy, S. A., Davies, R. M., & Li, H. (2021). Twelve years of SAMtools and BCFtools. GigaScience, 10(2), giab008. https://doi.org/10.1093/gigascience/giab008

Delaneau, O., Ongen, H., Brown, A. A., Fort, A., Panousis, N. I., & Dermitzakis, E. T. (2017). A complete tool set for molecular QTL discovery and analysis. Nature Communications, 8(1), Article 1. https://doi.org/10.1038/ncomms15452

Faust, G. G., & Hall, I. M. (2014). SAMBLASTER: Fast duplicate marking and structural variant read extraction. Bioinformatics, 30, 2503–2505. https://doi.org/10.1093/bioinformatics/btu314

Garrison, E. (2018). freebayes: Bayesian haplotype-based genetic polymorphism discovery and genotyping [C++]. https://github.com/ekg/freebayes (Original work published 2010)

Johnson, L. K., Brown, C. T., & Whitehead, A. (2019). Draft genome assemblies of killifish from the Fundulus genus with ONT and Illumina seqeuncing platforms. Zenodo.Langmead, B., & Salzberg, S. L. (2012). Fast gapped-read alignment with Bowtie 2 (Vol. 9). https://doi.org/10.1038/nmeth.1923

Li, H., Handsaker, B., Wysoker, A., Fennell, T., Ruan, J., Homer, N., Marth, G., Abecasis, G., Durbin, R., & Genome Project Data Processing, S. (2009). The Sequence Alignment/Map format and SAMtools. Bioinformatics, 25(16), 2078–2079. https://doi.org/10.1093/bioinformatics/btp352

Miller, J. T., Reid, N. M., Nacci, D. E., & Whitehead, A. (2019). Developing a High-Quality Linkage Map for the Atlantic Killifish Fundulus heteroclitus. G3 Genes|Genomes|Genetics, 9(9), 2851–2862. https://doi.org/10.1534/g3.119.400262

Monroe, J. G., Allen, Z. A., Tanger, P., Mullen, J. L., Lovell, J. T., Moyers, B. T., Whitley, D., & McKay, J. K. (2017). TSPmap, a tool making use of traveling salesperson problem solvers in the efficient and accurate construction of high-density genetic linkage maps. BioData Mining, 10, 38. https://doi.org/10.1186/s13040-017-0158-0

Purcell, S., Neale, B., Todd-Brown, K., Thomas, L., Ferreira, M. A. R., Bender, D., Maller, J., Sklar, P., de Bakker, P. I. W., Daly, M. J., & Sham, P. C. (2007). PLINK: A Tool Set for Whole-Genome Association and Population-Based Linkage Analyses. American Journal of Human Genetics, 81(3), 559–575.

Quinlan, A. R., & Hall, I. M. (2010). BEDTools: A flexible suite of utilities for comparing genomic features. Bioinformatics, 26(6), 841–842. https://doi.org/10.1093/bioinformatics/btq033

R Core Team. (n.d.). R: A language and environment for statistical computing. R Foundation for Statistical Computing, Vienna, Austria. https://www.R-project.org/

**Identification of species diagnostic loci**

To identify loci that exhibit fixed differences between *F. olivaceus* and all four clades of *F. notatus*, we constructed a geographically diverse list of individuals of each species from both reference and contact zone populations. Individuals selected from contact zones had Entropy proportion of ancestry (*q*) scores close to one for each species. The individuals selected were as follows:

*F. notatus*

Coss_119, Coss_125, Coss_143, Coss_159, Coss_168, Glov_031, Glov_092, Glover_35, Horse_10, Horse_45, Horse_48, Pascagoula_72, Pascagoula_73, Pascagoula_75, Pascagoula_76, Pascagoula_80, Pascagoula_81, Pascagoula_83, Pascagoula_85, Pascagoula_87, Pascagoula_94, Pascagoula_95, Pascagoula_97, Sabine_16, Sabine_35, Sabine_56, Sabine_1, Sabine_34, Sabine_36, Sabine_41, Sabine_54, Saline_17_10, Saline_17_49, Saline_17_5, Saline_17_89, Spr17_019, spr17_025, spr17_034, Spr17_040, Spr17_067, Spr17_105, Spr17_107, Spr17_110, Tom17_005, Tom17_016, Tom17_017, Tom17_061, Tom17_164, Tom17_168, Tomb17_102, Tomb17_64, Tomb17_96, Tomb17_99, BBH_15_3, Col_14_15, BBH_15_4, Cah_14_10, Red_Lit_3, Col_14_11, BBH_15_2, Col_14_12, Col_14_10, Red_Lit_2, Cah_14_13, Cah_14_12, BBH_15_5, CHO15_05, CHO15_06, Red_Lit_1, Angel_06, Angel_02

*F. olivaceus*

Coss_15_1, Coss_15_3, Coss_15_4, Coss_15_5, Horse_102, Horse_104, Horse_25, Horse_5, Horse_86, Horse_91, Noxubee_54, Noxubee_58, Noxubee_59, Pascagoula_1, Pascagoula_11, Pascagoula_14, Pascagoula_15, Pascagoula_16, Pascagoula_17, Pascagoula_18, Pascagoula_19, Pascagoula_26, Pascagoula_27, Sabine_26, Sabine_27, Sabine_30, Sabine_31, Sabine_48, Sabine_55, Sabine_64, Sabine_71, Sabine_81, Saline_17_18, Saline_17_21, Saline_17_22, Saline_17_27, Saline_17_56, Saline_17_77, Saline_17_86, Spr17_005, spr17_013, Spr17_015, Spr17_060, spr17_079, spr17_083, Spr17_092, Tom17_059, Tom17_097, Tom17_121, Tom17_166, Tomb17_202, Tomb17_204, Tom17_208, Gas_13_79, Yel_14_7, StFran_14_2, Gas_13_71, StFran_14_7, StFran_14_3, StFran_14_1, StFran_14_5, Gas_13_75, StFran_14_6, Yel_14_2, StFran_14_4, Yel_14_4, StFran_14_8, Gas_13_78, Yel_14_6, Yel_14_5, StFran_14_9

Supplementary Figure 1: Plot of F*_ST_* for random loci between *F. olivaceus* and *F. notatus* at the Pascagoula contact zone site. Estimates of *F_ST_* were generated by Stacks using the kernel-smoothing algorithm. The positions of species-diagnostic fixed loci, utilized in genomic cline analyses, are indicated by red dots. Chromosomes fused in *F. notatus* (1,6), (9,15), (10,19), (14,20) are ordered together.


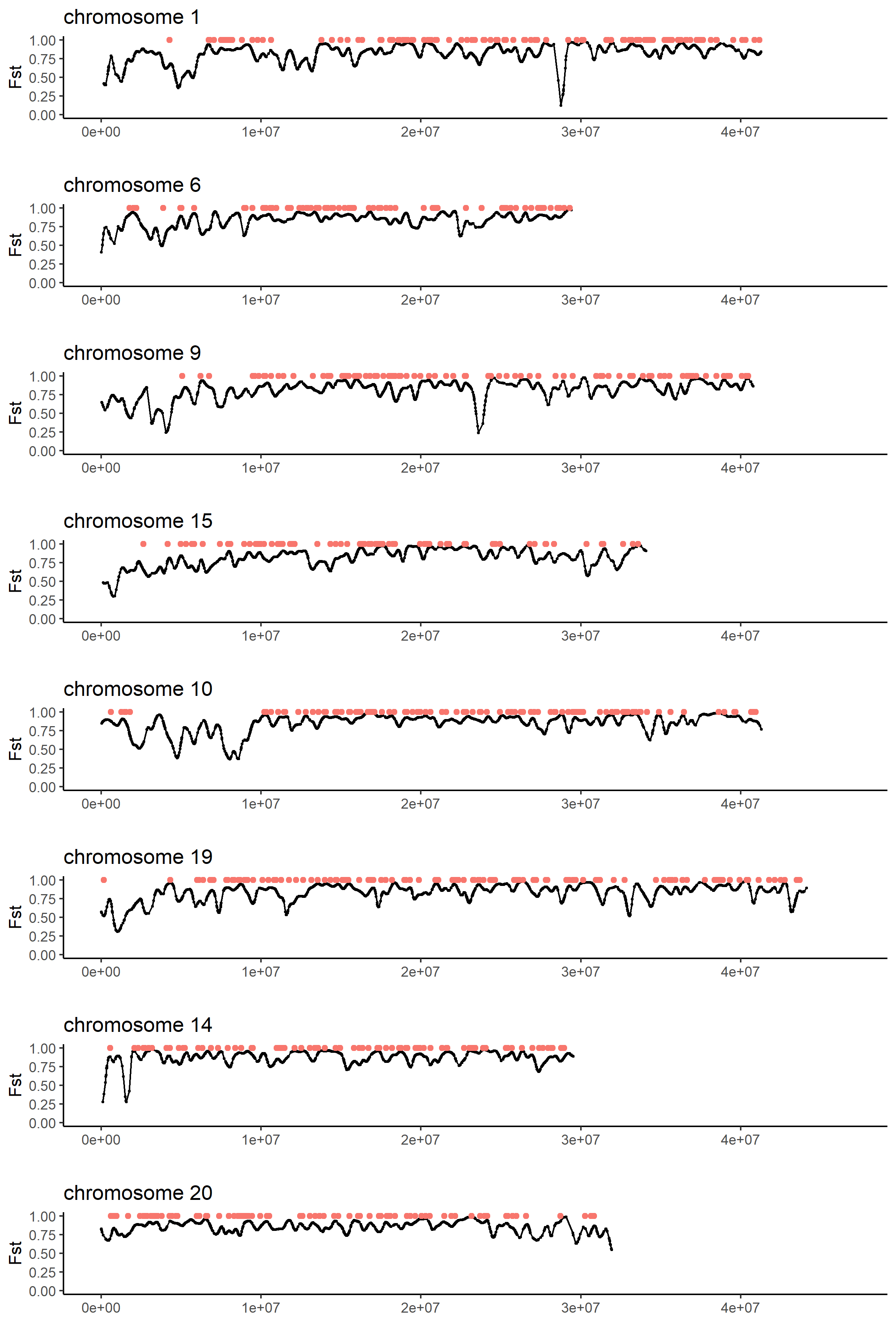


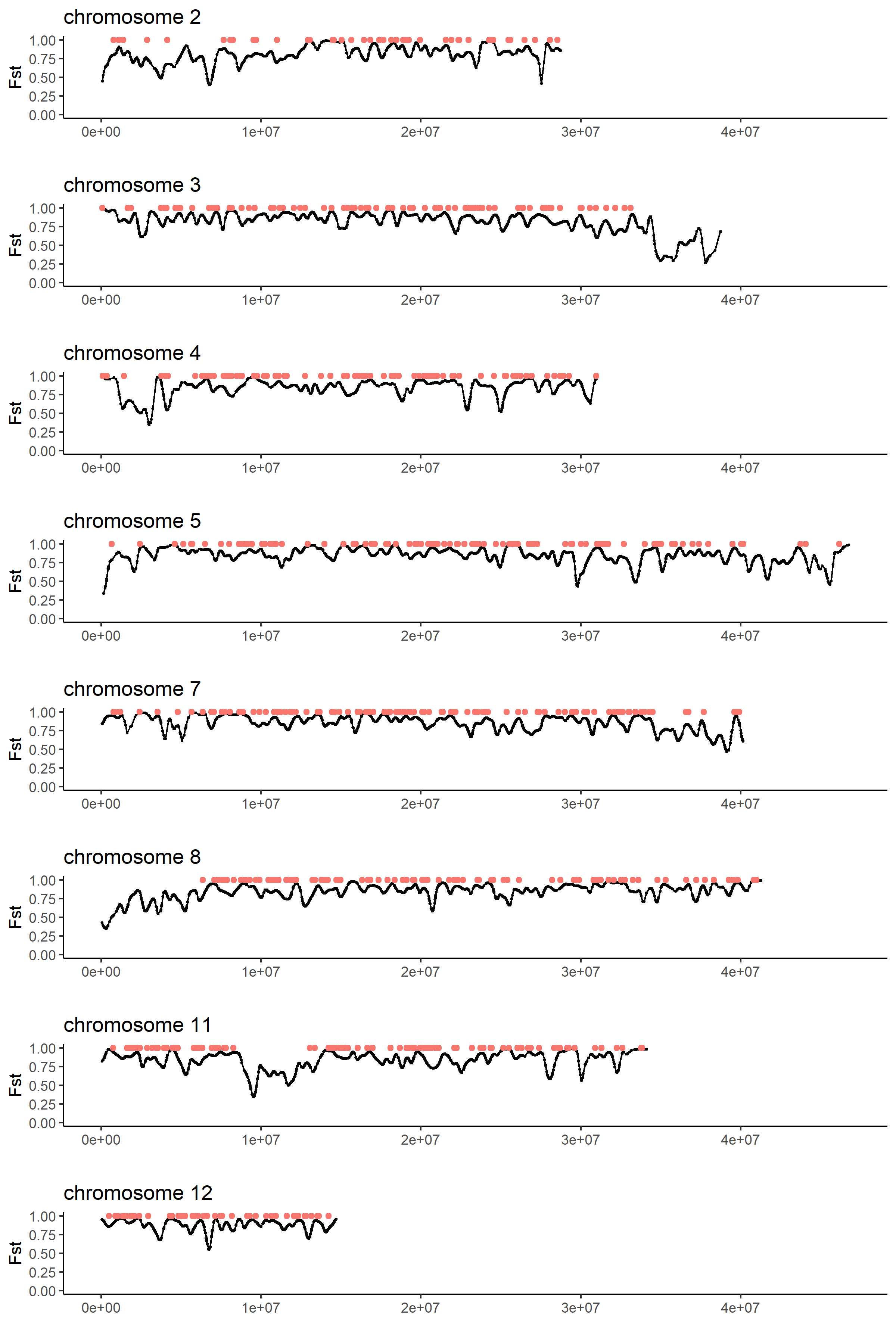


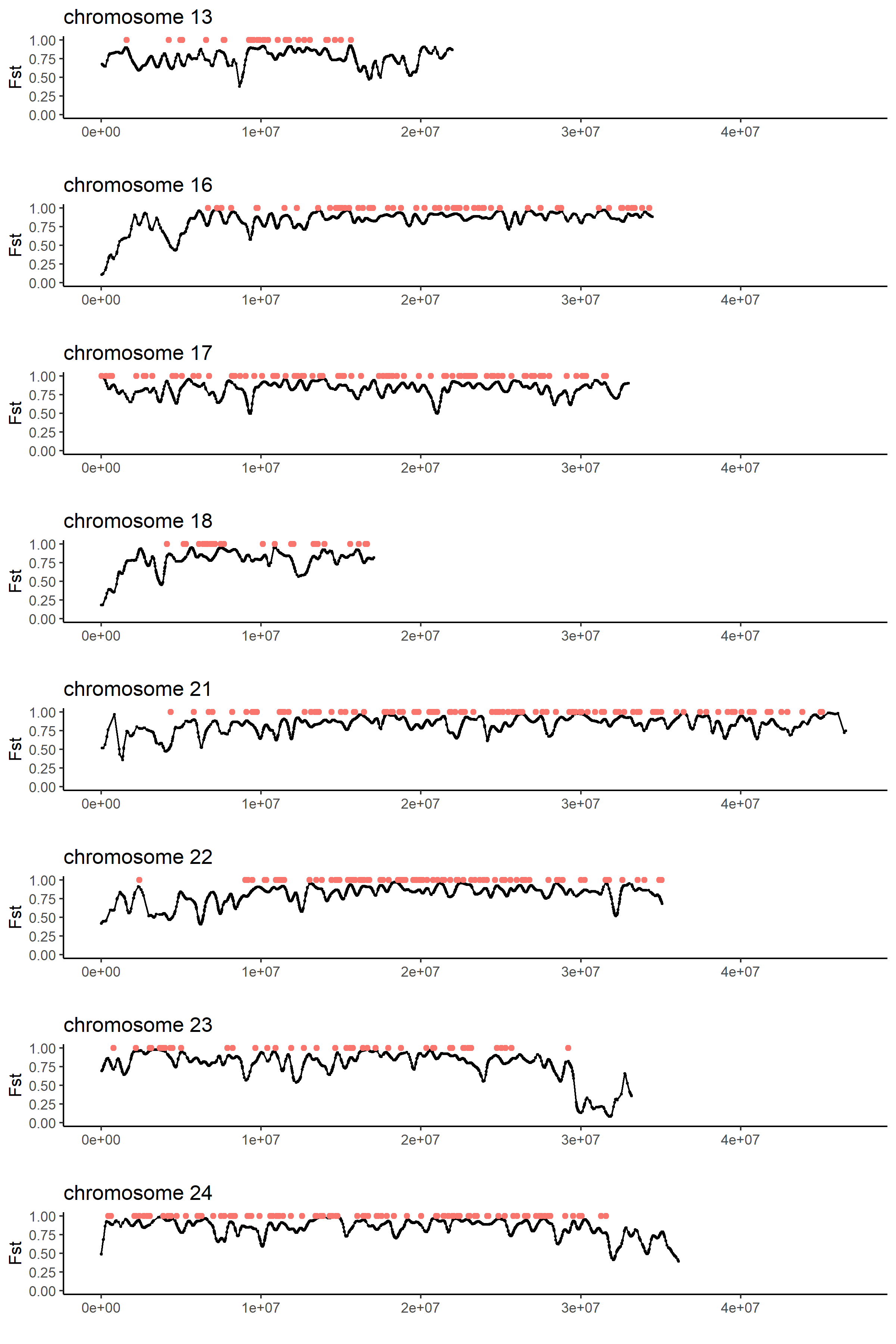


**Supplementary Figure 2:** Principle Coordinate Analysis of A) random loci and B) fixed loci. Symbol colors denote geographic assignment and shapes distinguish individual contact zones. Mississippi River basin orange (circle – Hor, triangle – Spr, diamond – Sal, square – Pas); Western Gulf Slope red (Sab); Red River basin green (circle – Cos, square – Glv); Mobile River basin yellow (circle – Nox, square – Tom). The species separate along the first axis with pure *F. notatus* on the left, *F. olicaceus* on the right, and putative F1 hybrids clustered near the middle.


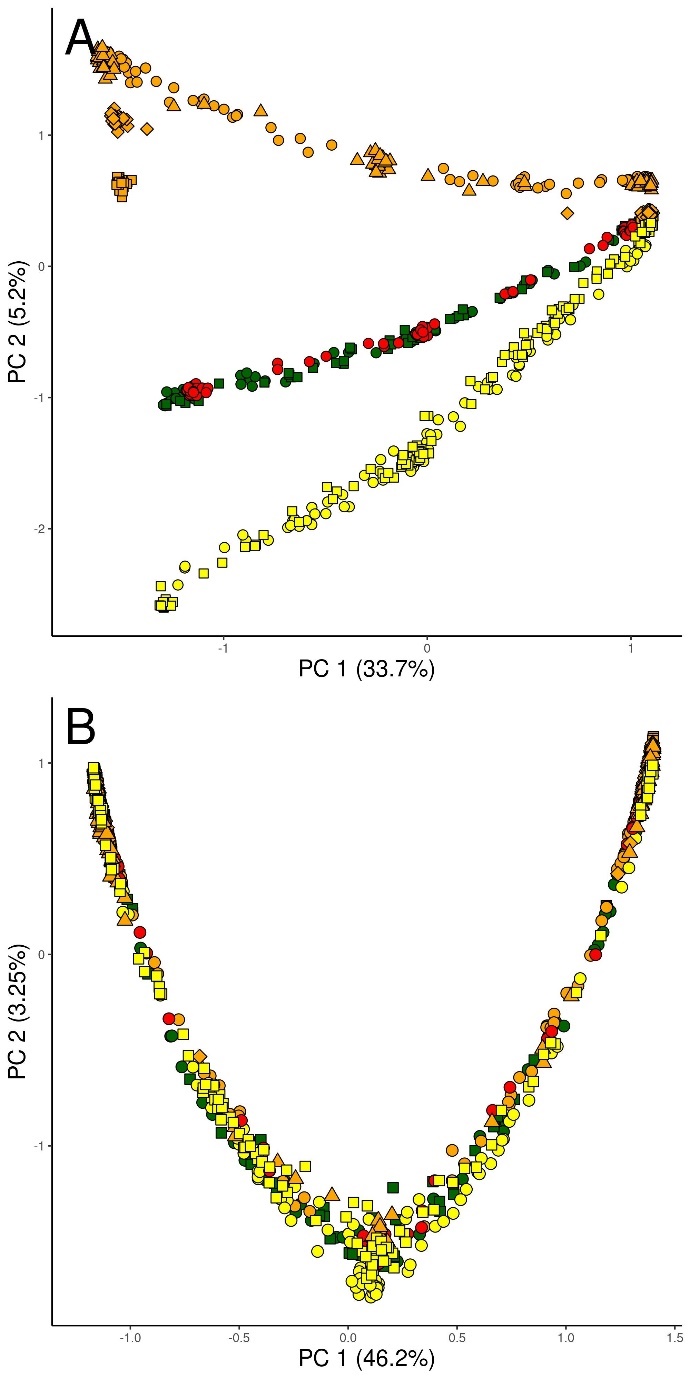

Supplement: Supplementary file 1 — Data S1 [file ECE3-13-e10399-s002.docx]
